# Supplementary material for: Identification of photoperiod-induced specific miRNAs in the adrenal glands of Sunite sheep (Ovis aries)
Source: Front Vet Sci. 2022 Jul 22;9:888207. doi: 10.3389/fvets.2022.888207 (PMC9354845; doi:10.3389/fvets.2022.888207)
Supplement: Supplementary file 1 [file Data_Sheet_1.ZIP › Supplementary file/miRDeep2 file.zip!/miRDeep2 file/Know_Structure/SP42A3/oar-mir-30d.pdf]

miRBase precursor : oar-mir-30d
Total read count : 237519
oar-miR-30d read count : 236727
remaining reads : 792

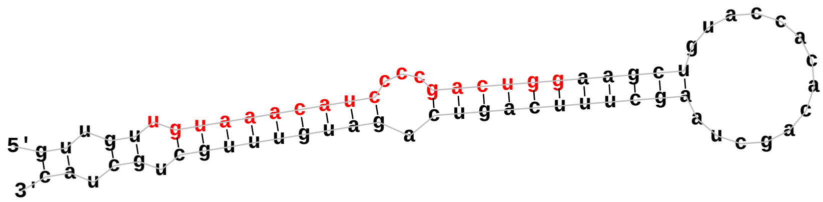

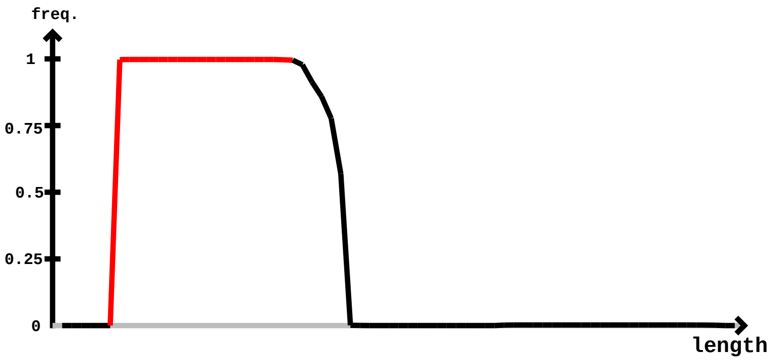


```
      oar-miR-30d
5'- guuguuguaaacauccccgacuggaagcuguaccacacagcuaagcuuucagucagauguuugcugcuac   -3'    exp
    ...((.(((((((((...(((((((((((..............))))))))))).))))))))).))...    reads    mm    sample
    ....uuguaaacauccccgacugga.............................................    1        0     seq
    ....uuguaaacauccccgacuggaagc..........................................    2        0     seq
    ....uuguaaacauccccgacuggaagcu.........................................    2        0     seq
    .....uguaaacaucccUgacu................................................    4        1     seq
    .....uguaaacauccccgacu................................................    235      0     seq
    .....uguaaacauccccgGcu................................................    2        1     seq
    .....uguaaacauccccgacug...............................................    401      0     seq
    .....uguaaacauccccgacugg..............................................    4057     0     seq
    .....uguaaacauccccgacugga.............................................    15344    0     seq
    .....uguaaacauccccgacuggaa............................................    13032    0     seq
    .....uguaaacauccccgacuggaag...........................................    19370    0     seq
    .....uguaaacauccccgacuggaagc..........................................    49300    0     seq
    .....uguaaacauccccgacuggaagcu.........................................    134849   0     seq
    .....uguaaacauccccgacuggaagcug........................................    175      0     seq
    .....uguaaacauccccgacuggaagcugu.......................................    75       0     seq
    .....uguaaacauccccgacuggaagcugua......................................    6        0     seq
    .....uguaaacauccccgacuggaagcuguacc....................................    2        0     seq
    .....uguaaacauccccgacuggaagcuguacca...................................    1        0     seq
    .....uguaaacauccccgacuggaagcuguaccaca.................................    2        0     seq
    ......guaaacauccccgacugga.............................................    1        0     seq
    ......guaaacauccccgacuggaa............................................    5        0     seq
    ......guaaacauccccgacuggaag...........................................    9        0     seq
    ......guaaacauccccgacuggaagc..........................................    30       0     seq
    ......guaaacauccccgacuggaagcu.........................................    75       0     seq
    .......uaaacauccccgacuggaagc..........................................    2        0     seq
    .......uaaacauccccgacuggaagcu.........................................    5        0     seq
    .........aacauccccgacuggaagcu.........................................    1        0     seq
    ...........................................agcuuucagucGgauguuu........    1        1     seq
    .............................................cuuucagucagauguuugc......    10       0     seq
    .............................................cuuucagucagauguuugcu.....    39       0     seq
    .............................................cuuucagucagauguuugcug....    27       0     seq
    .............................................cuuucagucagauguuugcugc...    127      0     seq
    .............................................cuuucagucagauguuugcugcu..    190      0     seq
    ..............................................uuucagucagauguuugcu.....    6        0     seq
    ..............................................uuucagucagauguuugcug....    6        0     seq
```

oar-miR-30d

```
guuguuguaaacauccccgacuggaagcuguaccacacagcuaagcuuucagucagauguuugcugcuac
..............................................uuucagucagauguuugcugc...    16        0          seq
..............................................uuucagucagauguuugcugcu..    105       0          seq
..............................................uuucagucagauguuugcugcua.    2         0          seq
..............................................uuucagucagauguuugcugcuac    1         0          seq
...............................................uucagucagauguuugcugc...    1         0          seq
```
